# Abundance, efficiency, and stability of reference transcript expression in a seasonal rodent: The Siberian hamster

**DOI:** 10.1371/journal.pone.0275263

**Published:** 2022-10-03

**Authors:** Calum Stewart, Timothy A. Liddle, Tyler J. Stevenson

**Affiliations:** School of Biodiversity, One Health and Veterinary Medicine, University of Glasgow, Glasgow, United Kingdom; Institute of Metabolic Science, University of Cambridge, UNITED KINGDOM

## Abstract

Quantitative PCR (qPCR) is a common molecular tool to analyse the expression of transcripts in non-traditional animal models. Most animals experience tissue-specific seasonal changes in cell structure, growth, and cellular function. As a consequence, the choice of reference or ‘house-keeping’ genes is essential to standardize expression levels of target transcripts of interest for qPCR analyses. This study aimed to determine the abundance, efficiency and stability of several reference genes commonly used for normalisation of qPCR analyses in a model of seasonal biology: the Siberian hamster (*Phodopus sungorus*). Liver, brown-adipose tissue (BAT), white adipose tissue (WAT), testes, spleen, kidney, the hypothalamic arcuate nucleus, and the pituitary gland from either long or short photoperiod Siberian hamsters were dissected to test tissue-specific and photoperiod effects on reference transcripts. qPCR was conducted for common reference genes including 18s ribosomal RNA (*18s*), glyceraldehyde 3-phosphate dehydrogenase (*Gapdh*), hypoxanthine-guanine phosphoribosyltransferase (*Hprt*), and actin-β (*Act*). Cycling time (Ct), efficiency (E) and replicate variation of Ct and E measured by percent coefficient of variance (CV%) was determined using PCR miner. Measures of stability were assessed using a combined approach of NormFinder and BestKeeper. *18s* and *Act* did not vary in Ct across photoperiod conditions. Splenic, WAT and BAT *Gapdh* Ct was higher in long compared to short photoperiod. Splenic *Hprt* Ct was higher in long photoperiods. There was no significant effect of photoperiod, tissue or interaction on measures of efficiency, Ct CV%, or efficiency CV%. NormFinder and BestKeeper confirmed that *18s*, *Gapdh* and *Hprt* were highly stable, while *Act* showed low stability. These findings suggest that *18s* and *Hprt* show the most reliable stability, efficiency, and abundance across the tissues. Overall, the study provides a comprehensive and standardised approach to assess multiple reference genes in the Siberian hamster and help to inform molecular assays used in studies of photoperiodism.

## Introduction

Quantitative PCR (qPCR) is one of the most used techniques for basic research and has been crucial for molecular analyses in many non-traditional animal models due to the specificity and sensitivity of the assay. qPCR is extremely important for studies that seek to understand the molecular basis of life history transitions including hibernation, migration, and photoperiodism. Traditionally, most studies have used hormone or protein-based assays to identify cellular or neural mechanisms that govern seasonal changes in physiology. However, molecular analyses have become increasingly common, both as the primary means of data analysis and as complementary data to traditional methods. qPCR analyses are one of the most precise forms of molecular analysis, yet the reliability of qPCR depends on the stability and reliability of reference transcripts.

There are multiple methods available to assess transcript expression levels based on PCR amplification assays, and PCR Miner provides a comprehensive and objective data analysis tool [1]. PCR Miner uses an algorithm based on exponential growth rate, providing an objective calculation of cycling time (Ct), the key measure used to determine transcript expression levels. Furthermore, the PCR Miner algorithm calculates both the efficiency of the amplification reaction and the coefficient of variation of replicates (within sample variability). In this way, PCR Miner produces an overall assessment of the PCR amplification and uses an open, reliable, and mathematically based approach for molecular analyses. Measures of transcript expression stability are also important to ensure high quality reference genes are selected and two different software approaches commonly used are BestKeeper [2] and NormFinder [3]. NormFinder produces a stability value in which more stable expression is associated with values closer to 0. Alternatively, BestKeeper produces a coefficient of variation (r) and more stable reference expression is closer to 1.0. By using a combination of multiple stability values, efficiency measures and percent coefficient of variation, reference genes can be ranked according to which ones are the most suitable for qPCR analyses.

Most mammals living in temperate and equatorial zones exhibit remarkable seasonal changes in growth and metabolism [4]. The underlying changes in cell structure and function raises a major challenge for the selection of stable and robust reference transcripts for qPCR analyses. Across plants and animals, several reference transcripts are consistently used such as Ribosomal RNA which are protein-synthesizing molecules in ribosomes. Several ribosomal RNAs are present in prokaryotes and eukaryotes and generally differentiated based on size, or svedberg unit (S, e.g., 18s) [5]. In birds and mammals, other reference transcripts have been selected including β-actin (*Act*), glyceraldehyde 3-phosphate dehydrogenase (*Gapdh*), and hypoxanthine-guanine phosphoribosyltransferase (*Hprt*) [6, 7]. It is generally assumed that reference transcripts have constant expression levels across control and experimental conditions with most studies reporting simple analyses of average Ct [8]. However, several reports investigating the impact of photoperiod on target transcript levels also described effects on reference transcript levels in thale cress *Arabidopsis* (*A*. *thaliana;* [9]), forest trees *Populus* (*P*. *trichocarpa* and *P*. *tremula*; [10]), Sugarcane [11] and Brandt’s voles (*Lasiopodomys brandtii;* [7]). Consequently, the Ct, efficiency and stability of multiple reference transcripts should be considered in studies that incorporate photoperiod manipulations.

Siberian hamsters are a commonly used animal model for the molecular basis of mammalian photoperiodism [12]. qPCR analyses are a common method used to assess photoperiod-dependent molecular changes in Siberian hamsters. Reference genes *18s* [13–15], *Act* [16–20], *Gapdh* [15, 18, 19, 21], *Hrpt* [14, 15, 17] and cyclophilin A [22] are frequently used reference genes within such analyses. To our knowledge, no study has systematically assessed the suitability of multiple reference genes across tissues and photoperiodic treatment.

This study aimed to determine the abundance, efficiency, and stability of four reference genes across eight tissues in an animal model of seasonality: Siberian hamster. Hamsters were collected from either long day, summer-like photoperiod or short day, winter-like photoperiod, and qPCRs for *18s*, *Act*, *Hprt*, and *Gapdh* conducted on liver, white adipose tissue (WAT), brown adipose tissue (BAT), kidney, spleen, testes, pituitary, and the arcuate nucleus in the hypothalamus. These tissues were selected due to the role in the control of seasonal changes in energy and water balance, reproduction, and immune function. We hypothesised that there will be tissue- and photoperiod-specific effects on reference transcript abundance, efficiency, and stability. However, *18s* and *Hprt* amplification and expression was consistent across photoperiod treatment, and most tissues. *18s* and *Hprt* were also found to be the most stable reference genes. Photoperiod effects on transcript abundance was only identified for *Gapdh* in spleen, brown- and white-adipose tissue, and splenic *Hprt*. Our data provide a resource to improve qPCR analyses that use photoperiod as a measure in studies investigating mechanisms of seasonal biology.

## Methods

### Animals and ethical permissions

Adult male hamsters (3–8 months) were obtained from a colony maintained at the University of Glasgow. Hamsters were raised in polypropylene cages illuminated for 16h of light and 8h of darkness per day. Harlan food and tap water were provided *ad libitum* and each cage was provided cotton-nesting material. All procedures were approved by the University of Glasgow Animal Welfare and Ethics Committee and Home Office approved (PPL PP5701950). All procedures were in accordance with the Arrive Guidelines for ethical research on animals.

### Experimental design

Male Siberian hamsters were held under either long photoperiod (LD) (16h light:8h dark) (n = 6) or short photoperiod (SD) (8h light:16h dark) (n = 6) for 12 weeks. Animals were age matched across the two photoperiod treatments to remove potential confound of hamster age. Hamsters maintained in long photoperiod had testes (avg. 0.73g +/-0.04g SEM) and body mass (avg. 41.2g +/-1.3g SEM) measurements indicative of the summer breeding state. All hamsters collected after short photoperiod manipulation showed involution of the gonads (avg. 0.07g +/-0.004g SEM) and body mass (avg. 35.1g +/-0.8g SEM) which reflect the photoregressed state. Animals were then sacrificed by cervical dislocation followed by exsanguination. Tissues (Liver, WAT, BAT, Kidney, spleen, testes, pituitary, and brain) were immediately dissected, frozen on dry-ice and stored at -80°C until RNA extraction. All dissections were conducted between 1.5 hours after lights on and were complete within 3 hours (i.e., zeitgeber time 1.5–4.5h). The hamster brain was sectioned into 200 μm coronal sections using a Leica cryostat. Tissue sections with anatomical structures including optic tract to the infundibular stem (approximately -2.12mm to -3.80mm from Bregma) [23] were used to isolate the arcuate nucleus. Bilateral tissue punches were performed using an integra Miltex 1mm disposable biopsy punch. Confirmatory PCR analyses for somatostatin were conducted to ensure tissue specificity.

### RNA extraction and cDNA synthesis

RNA was extracted from tissues using RNeasy Plus Mini kit (Qiagen) as per manufacturer’s instructions. RNA quantity and purity were measured using a Nanodrop ND-1000 (NanoDrop Technologies) and used in cDNA synthesis procedure. cDNA synthesis reaction mixture contained 4 μl 100ng/μl total RNA (400 ng total), 2 μl 5X first strand buffer (Thermofisher Scientific), 1 μl DTT (10mM), 0.2 μl 20mM Random Primers (Promega), 0.2 μl 20mM dNTP mix (Thermofisher Scientific), 0.26 μl RNasin® Ribonuclease Inhibitor (Promega), 0.26 μl Superscript III reverse transcriptase (Thermofisher Scientific), 2.08 μl RNAse free water. Reaction mixture was incubated at 50°C for 1 hour. Once incubation was complete mixture was diluted with 90 μl LOTE buffer (3mM Tris-HCL (Thermofisher Scientific), 0.2 mM EDTA(Sigma)) and cDNA was stored at −20°C until qPCR. Quantification of cDNA was achieved using Agilent Brilliant II SYBR green. All samples were run in duplicate in using an Agilent Stratagene MX300p with the following conditions: 1) denaturing: 95°C for 5 minutes; 2) Cycling: 40 times through a 95°C denature for 30 seconds, an annealing temperature for 1 minute that was primer specific (See Table 1), and an extension period set at 72°C for 30 seconds. A melting curve assays was included after the PCR amplification and consisted of increasing from 55°C for 30 seconds to 95°C and fluorescence measured at each temperature. A single peak in fluorescence was used to confirm specificity of amplification. PCR Miner was used to determine the cycle thresholds, reaction efficiencies and variability in replicate amplification (i.e., % coefficient of variation).

**Table 1:** Primer sequences and qPCR parameters.

Gene	Primer	Size	Temp	Melt
*18s*	GCTCCTCTCCTACTTGGATAACTGTG	111	62˚C	80˚C
	CGGGTTGGTTTTGATCTGATAAATGCA			
*Gapdh*	TTCTTGTGCAGTGCCAGCCTCG	207	60˚C	85˚C
	CTGTGCCGTTGAACTTGCCGTG			
*Hrpt*	AGTCCCAGCGTCGTGATTAGTGATG	141	62˚C	76˚C
	CGAGCAAGTCTTTCAGTCCTGTCCA			
*Act*	CTGGAACGGTGAAGGTGACA	63	60˚C	84˚C
	AAGGGACTTCCTGTAACAATGCA			

### Statistical analyses

A general linear model was used to assess the main effects of photoperiod and tissue on cycling time, efficiency and coefficient of variation using SigmaPlot 14.0. Kolmogorov-Smirnov test were conducted to determine normal distribution of raw data. In the event there was a violation of normality, data were log-transformed. Fisher’s least significant different (LSD) method was used to determine pairwise differences when a significant interaction was established. All analyses passed the Brown-Forsythe test of equal variances. The level of statistical significance was set at p < 0.05. Transcript expression stability was evaluated using the programs NormFinder (https://moma.dk/normfinder-software), and BestKeeper (https://www.gene-quantification.de/bestkeeper.html). NormFinder program provides a stability value number for each gene, lower stability values indicating less stability [2]. The BestKeeper program calculates a Pearson’s correlation coefficient for each gene, values of p closer to 1.0 indicating greater stability [3].

## Results

The raw results for all transcript analyses are available in S1 Table. Overall, expression abundance was highest for *18s*, followed by *Gapdh*, *Hprt* and the lowest expressed gene was *Act*. Average efficiencies of the reactions were all within the MIQE guidelines [24]. *Act* had low expression stability, while *18s*, *Gapdh* and *Hprt* were stably expressed across tissues.

### Reference gene abundance under long and short photoperiods

**Fig 1:**
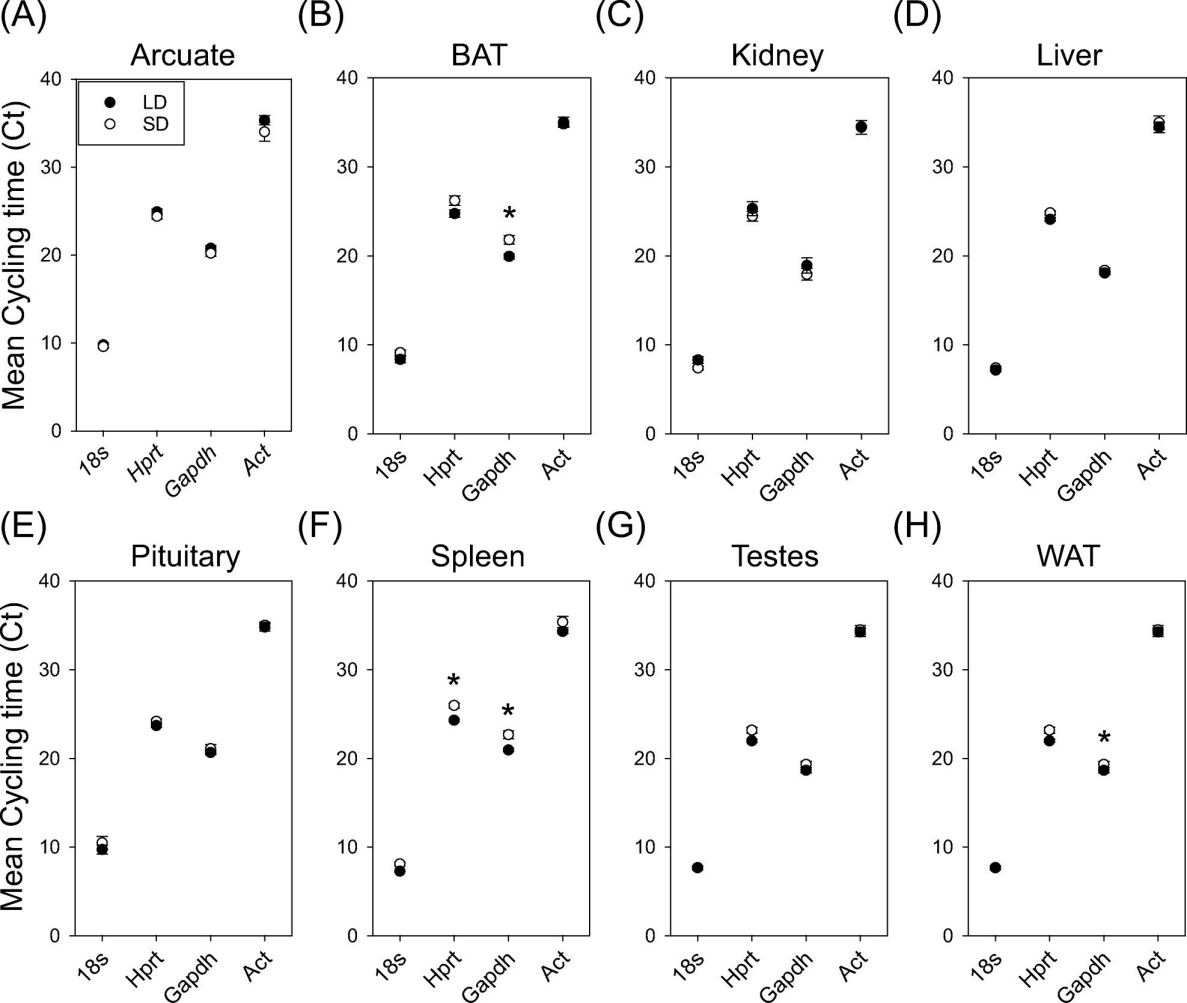
Abundance of 18s, Hprt, Gapdh and Act in hamster tissues across photoperiod conditions. Mean Cycling time (Ct) of 18s, Hprt, Gapdh and Act in the arcuate nucleus (A), brown adipose tissue (BAT) (B), kidney (C), liver (D), pituitary (E), spleen (F), testes (G) and white adipose tissue (WAT) (H). Asterix indicates pairwise significant difference at P < 0.05.

There was no significant main effect of photoperiod on *18s* expression (F_1, 77_ = 0.45; P = 0.50). But there was a significant effect of tissue on *18s* expression (F_7, 77_ = 17.33; P < 0.01). There was no significant interaction for photoperiod and tissue on expression (F_7, 77_ = 1.91; P = 0.07) (Fig 1). There was a significant main effect of photoperiod (F_1, 77_ = 4.03; P < 0.05) and tissue (F_7, 77_ = 8.14; P < 0.01) on *Hprt* expression. There was a significant interaction of photoperiod and tissue on *Hprt* expression (F_7, 77_ = 2.18; P = 0.04). Fishers LSD pairwise comparison revealed splenic *Hprt* levels were significantly lower in SD compared to LD photoperiod (Fig 1F, P = 0.02). There was no effect of photoperiod on *Gapdh* expression (F = 1.50 _1, 77_; P = 0.22). But there was a significant effect of tissue on *Gapdh* expression (F_7, 77_ = 12.68; P < 0.001). There was a significant interaction of photoperiod and tissue on *Gapdh* expression (F_7, 77_ = 2.45; P = 0.025). Fishers LSD pairwise comparison test for *Gapdh* identified significantly lower levels in SD spleen (Fig 1F, P = 0.03), BAT (Fig 1B, P = 0.02), and WAT (Fig 1H, P = 0.03), compared to LD levels. *Act* amplification was insufficient for analyses in 13 samples (S1 Table) and were removed from the analyses. There was no significant effect of photoperiod (F_1, 64_ = 1.03; P = 0.31) or tissue (F_7, 64_ = 0.22; P = 0.98) on *Act* expression. There was no interaction of photoperiod and tissue on *Act* expression (F = 0.49 _7, 64_; P = 0.84).

### Lack of Ct variability in reference transcript amplification

**Fig 2:**
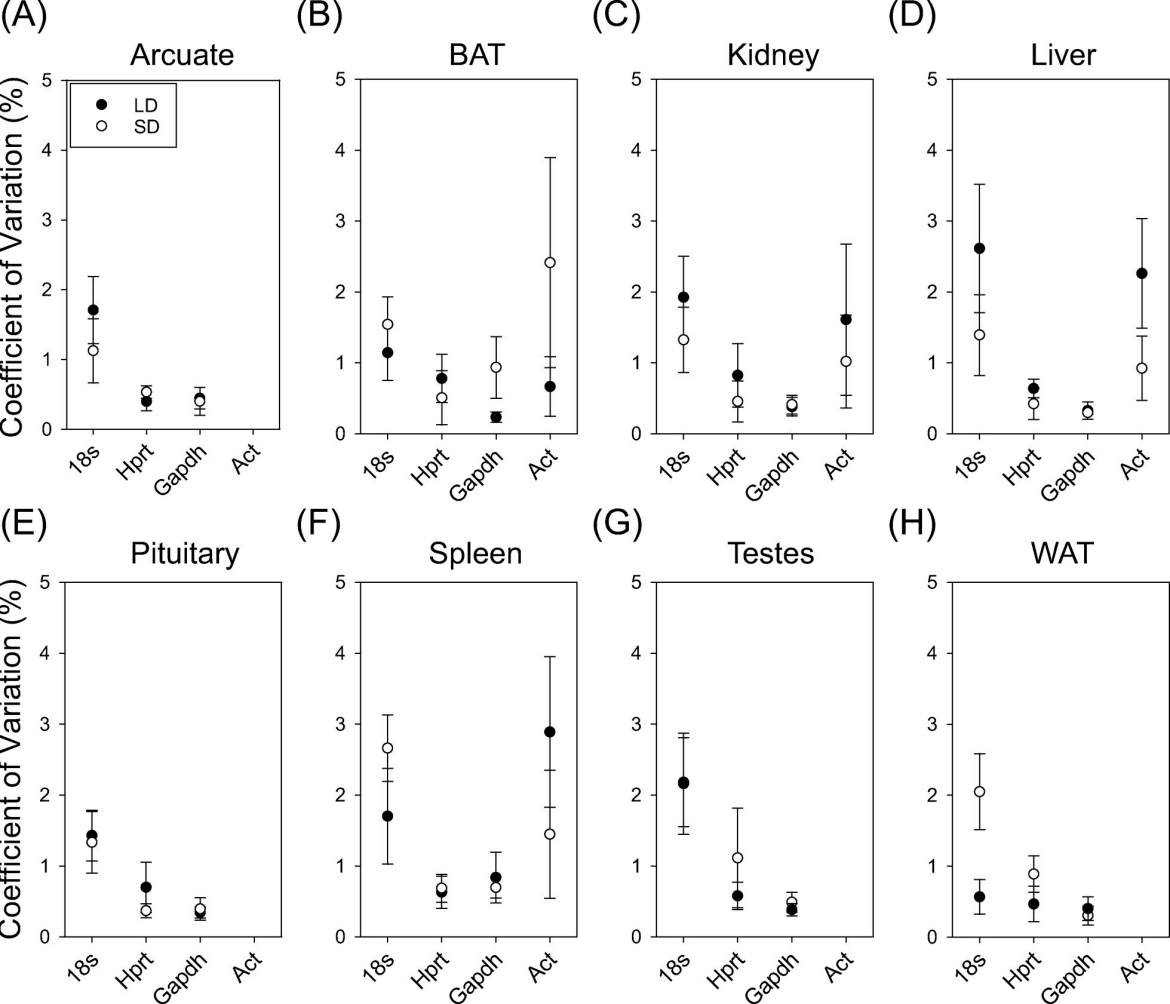
Coefficient of variation of Ct of 18s, Hprt, Gapdh and Act in hamster tissues across photoperiod conditions. Mean coefficient of variation (CV%) of 18s, Hprt, Gapdh and Act in arcuate (A), BAT (B), kidney (C), liver (D), pituitary (E), spleen (F), testes (G) and Wat (H).

Next, we assessed the level of variability within in the reference transcript amplification for the duplicates amplified in each hamster sample. replicates using PCR Miner percent coefficient of variation values (Fig 2A–2H). The precent coefficient of variation is an indication of the amount of deviation in the two cycling times and a lower value is associated greater similarity in replicate amplification. There was no significant effect of photoperiod (F_1, 76_ = 3.04; P = 0.08), tissue (F_7, 76_ = 0.87; P = 0.53) or interaction on *18s* variability (F_7, 76_ = 1.66; P = 0.13). There was also no significant effect of photoperiod (F_1, 76_ = 1.22; P = 0.27), or tissue (F_7, 77_ = 0.13; P = 0.99), or significant interaction on *Hprt* variability (F_7, 77_ = 0.80; P = 0.59). Likewise, there was no significant effect of photoperiod (F_1, 77_ = 0.86; P = 0.35), or tissue (F = 0.88 _7, 77_; P = 0.52), or interaction of photoperiod and tissue on *Gapdh* (F_7, 77_ = 0.53; P = 0.81). Finally, there was no significant effect of photoperiod (F_1, 23_ = 0.21; P = 0.65), or tissue (F_3, 23_ = 1.11; P = 0.37) or significant interaction of photoperiod and tissue on *Act* Ct variability (F_3, 23_ = 0.74; P = 0.54). Due to poor amplification, we removed the *Act* percent coefficient of variation for arcuate, pituitary, testes, and WAT tissues. Altogether, these data indicate that the expression levels of the reference transcripts using the primer sequences produced high inter-replicate reliability.

### Reference transcript amplification efficiency

**Fig 3:**
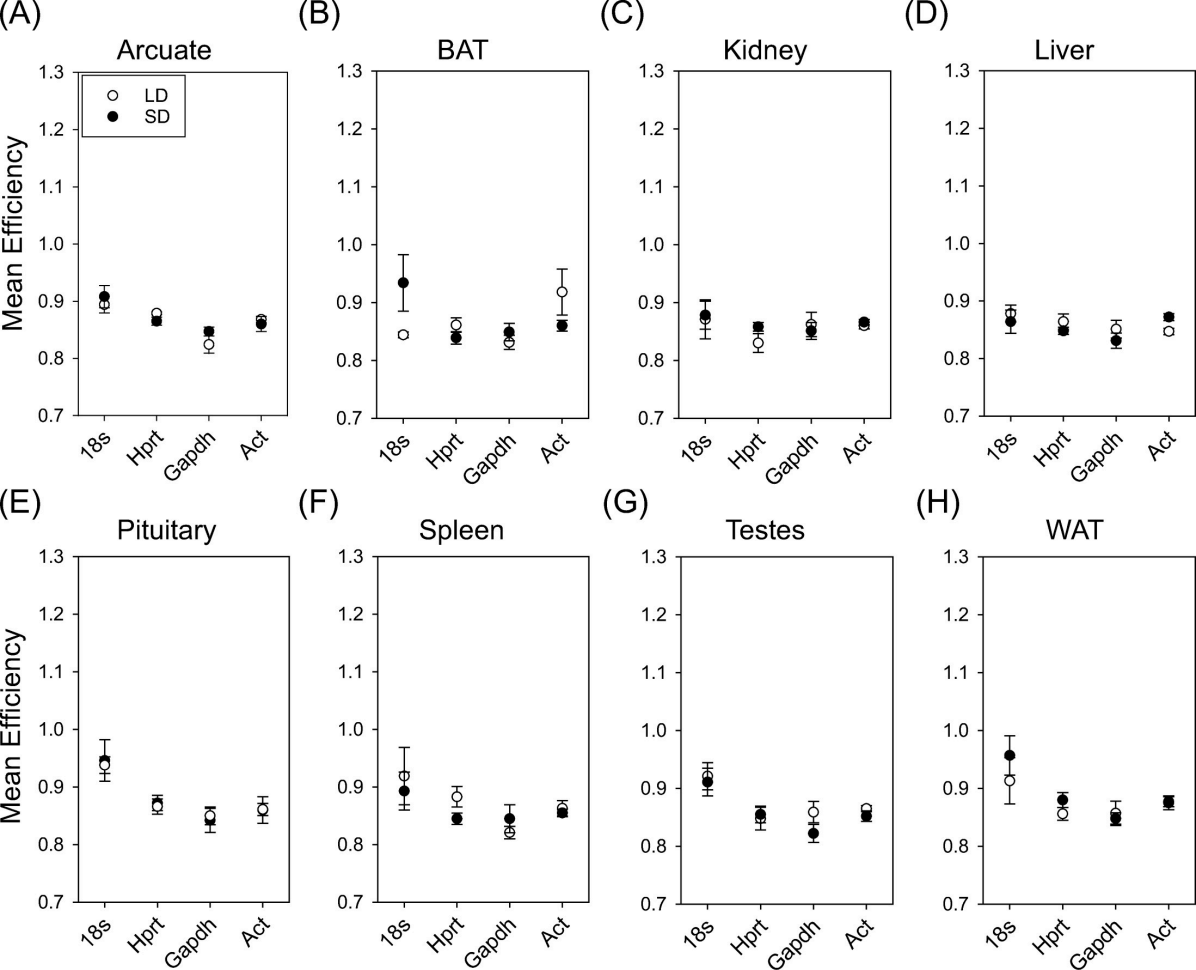
Expression efficiency of 18s, Hprt, Gapdh and Act in hamster tissues across photoperiod conditions. Mean coefficient of variation (CV%) of 18s, Hprt, Gapdh and Act in arcuate (A), BAT (B), kidney (C), liver (D), pituitary (E), spleen (F), testes (G) and Wat (H).

Next, we assessed the efficiency of each reference transcript primer pair across photoperiod and tissues (Fig 3A–3H). Overall, there was remarkable consistency in reference transcript efficiencies. There was no significant effect of photoperiod (F_1,77_ = 0.04; P = 0.83), or tissue (F_7,77_ = 1.39; P = 0.22), or interaction of tissue and photoperiod on *18s* efficiency (F_7,77_ = 0.23 P = 0.98). Moreover, there was no significant effect of photoperiod (F_1,77_ = 0.62; P = 0.43), or tissue (F_7,77_ = 1.34; P = 0.24), or interaction of tissue and photoperiod on *Hprt* efficiency (F_7,77_ = 1.69 P = 0.12). There was also no significant effect of photoperiod (F_1,77_ = 0.01; P = 0.94), or tissue (F_7,77_ = 0.47; P = 0.85), or interaction of photoperiod and tissue on *Gapdh* efficiency (F_7,77_ = 0.94; P = 0.48). Lastly, there was no significant effect of photoperiod (F_1,64_ = 0.29; P = 0.59), or tissue (F_7,64_ = 1.00; P = 0.43) or interaction of photoperiod and tissue on *Act* efficiency (F_7,64_ = 0.1.06; P = 0.40). These data confirm that the amplification of each reference transcript was similar across photoperiodic treatments and tissues.

### Low inter-replicate variability in reference transcript efficiency

**Fig 4:**
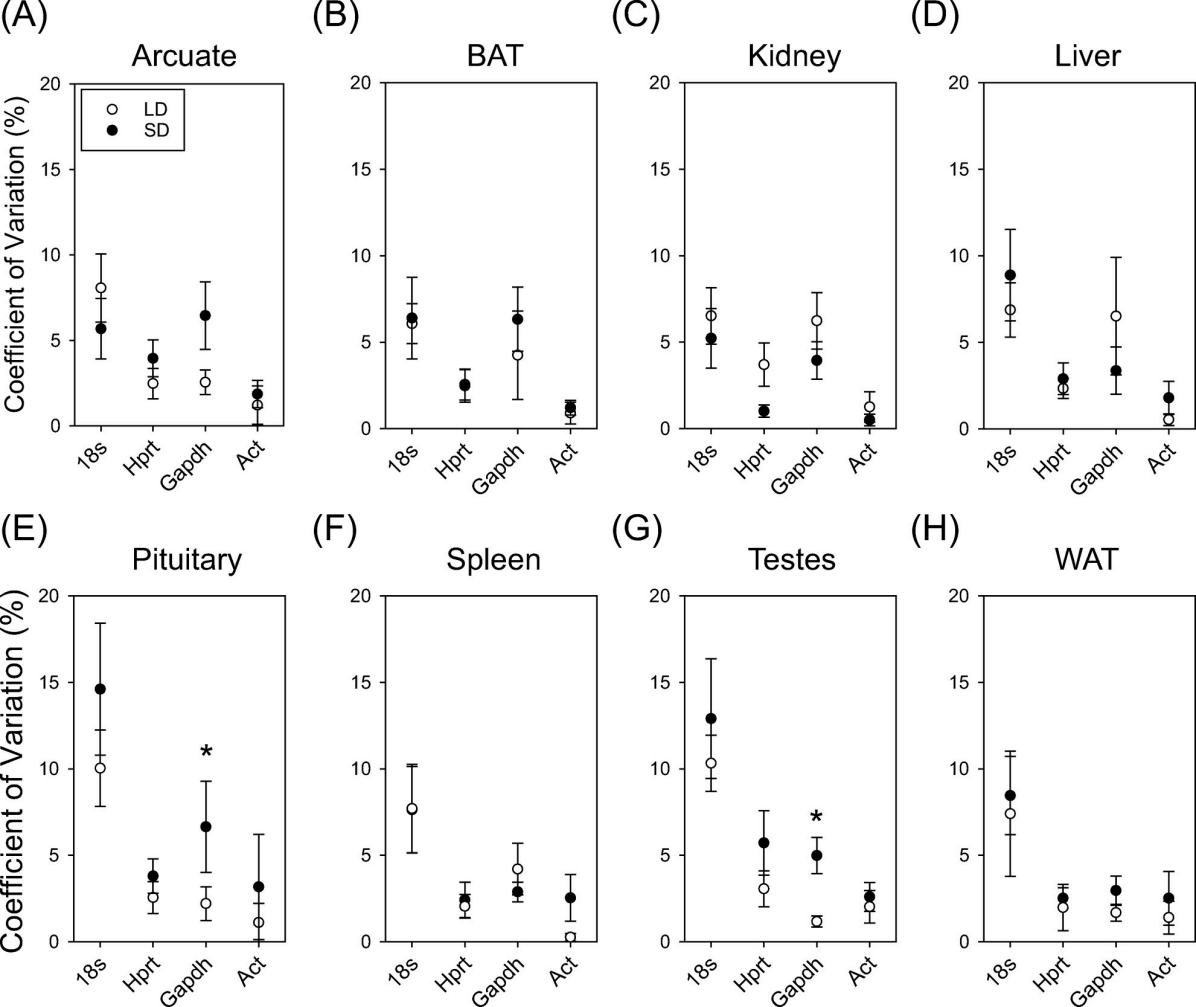
Coefficient of variation of expression efficiency of 18s, Hprt, Gapdh and Act in hamster tissues across photoperiod conditions. Mean coefficient of variation (CV%) of 18s, Hprt, Gapdh and Act in arcuate (A), BAT (B), kidney (C), liver (D), pituitary (E), spleen (F), testes (G) and Wat (H). Asterix indicates pairwise significant difference at P < 0.05.

To examine variability across replicates, we assessed the efficiency coefficient of variation (Fig 4A–4H). There was no significant effect of photoperiod (F_1,77_ = 0.00; P = 0.97), or tissue (F_7,77_ = 1.94; P = 0.07) or interaction of tissue and photoperiod on *18s* efficiency coefficient of variation (F_7,77_ = 0.49; P = 0.83). Likewise, there was no significant effect of photoperiod (F_1,77_ = 0.28; P = 0.60), or tissue (F_7,77_ = 0.95; P = 0.47), or interaction of tissue and photoperiod on *Hprt* efficiency (F_7,77_ = 1.098; P = 0.37). There was also no significant effect of photoperiod (F_1,77_ = 0.02; P = 0.89), or tissue (F_7,77_ = 0.62; P = 0.73). There was a significant interaction of photoperiod and tissue on the percent coefficient of variation in *Gapdh* efficiency (F_7,77_ = 2.19; P = 0.04). Fishers LSD pairwise comparison test revealed that pituitary and testes *Gapdh* coefficient of variation for efficiency was more variable within replicates in SD compared to LD tissue (Fig 4E, P = 0.009; Fig 4G, P = 0.03). Lastly, there was no significant effect of photoperiod (F_1,26_ = 0.35; P = 0.56), or tissue (F_7,26_ = 0.66; P = 0.70) or interaction of photoperiod and tissue on *Act* (F_7,64_ = 1.09; P = 0.39). These findings establish that *18s*, *Hprt* and *Act* maintain low variability in amplification efficiency across photoperiod and tissue samples.

### Reference transcript expression stability

**Fig 5:**
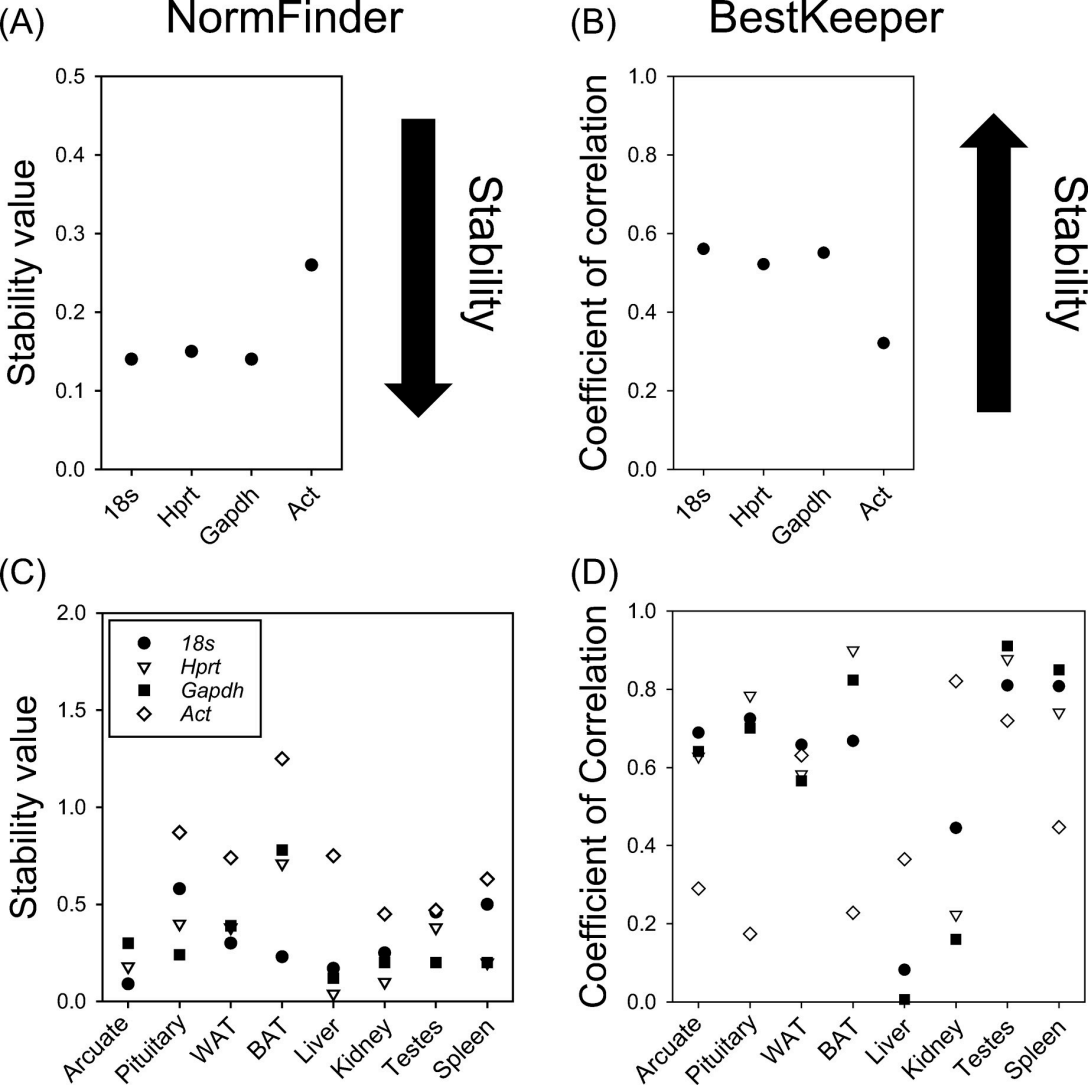
Stability of 18s, Hprt, Gapdh and Act expression across multiple hamster tissues. Stability in reference transcript levels across multiple tissues using NormFinder (A). 18s, Hprt, and Gapdh show high stability while Act has lower stability across all tissues. Note that lower NormFinder values represent higher stability. Stability in reference transcripts across tissued based on Bestkeeper (B). 18s, Hprt, and Gapdh show high stability while ACT has lower stability. Note that higher BestKeeper values represent higher stability. Tissue specific effects on photoperiod stability of 18s, Hprt, Gapdh and Act using Normfinder (C). Stability of 18s, Hprt, and Gapdh across photoperiods remains high in all tissues while Act shows lower stability. Tissue specific stability of 18s, Hprt, Gapdh and Act across photoperiods using Bestkeeper (D). Stability of 18s, Hprt, and Gapdh remains high in most tissues, Act has highest stability in the pituitary gland.

Finally, we assessed the expression stability of genes across tissues and photoperiod using NormFinder and Bestkeeper. Normfinder showed that *18s*, *Gapdh* and *Hprt* had high levels of stability (stability value < 0.2), however *Act* had low levels of stability (stability value > 0.2) (Fig 5A). Likewise, Bestkeeper found that *18s*, *Gapdh* and *Hprt* had high levels of stability, however *Act* had low levels of stability (Fig 5B). Stability of transcripts across photoperiod conditions was then assessed within individual tissues. Normfinder revealed that *18s*, *Gapdh* and *Hprt* remained highly stable in most tissues (Fig 5C). Bestkeeper revealed that *18s*, *Gapdh* and *Hprt* were stable across most tissues, except for liver and kidney where they showed lower stability, *Act* showed stability in only kidney (Fig 5D). These findings suggest that stability for reference genes *18s* and *Hprt* is maintained across tissues in Siberian hamsters.

## Discussion

In this report we have identified that *18s* and *Hprt* maintain high stability across photoperiodic conditions in the Siberian hamster. These reference genes, therefore, may represent suitable choices for investigations of photoperiodism in Siberian hamsters. However, *Hprt* was found to show photoperiod-dependent regulation of expression levels in splenic tissue, and consequently, other reference genes should be selected. *Gapdh*, while slightly less stable, also displayed significant photoperiod driven differences in BAT, WAT and spleen expression levels. *Act* was the least stable of genes investigated, however may be suitable to construct geometric means for some molecular studies due to constant expression levels across photoperiod treatments. These data provide a tissue specific ranking system for reference transcripts used in qPCR analyses of Siberian hamsters (S1 Table) and the ability to establish a molecular dataset to help standardize assays across laboratories.

Photoperiod effects on reference transcript abundance (i.e., Ct) and variability within replicates is a major confound for normalization of target transcript of interest analyses. Therefore, it is essential that reference transcripts maintain constant levels across photoperiod treatments. Here, we report that *Gapdh* expression in the spleen, BAT and WAT is regulated by photoperiod, with increased levels in short photoperiods. The consequence of *Gapdh* abundance to normalize expression levels would result in higher fold change in short photoperiod samples and confound any interpretation. In Siberian hamsters, *18s* and *Act* expression abundance was consistent across photoperiods. However, the stability of *Act* is poor across photoperiod treatments for most tissues. Instead, *18s* and *Hprt* are reliably the most stable. Effects of photoperiod on reference transcript expression levels and stability are also reported in Thale cress (*A*. *thaliana*, [9]), sugarcane [11], Populus [10], and Brandt’s voles [7]. In *A*. *thaliana*, a commonly used model of plant photoperiodism, most reference transcripts such as *Act* ubiquitin extension protein (*UBQ1*) and elongation factor-1 alpha (*EF1α*) in seedlings maintain abundance levels across photoperiod treatments [9]. However, only *Act* had high stability across photoperiod conditions [9]. In sugarcane, *UBQ1* was highly stable across photoperiods, but *Gapdh* was less stable [11]. Populus species, *18s* stability by Normfinder is highly dependent on the tissue investigated [10]. In Brandt’s voles, hypothalamic *Act* and peptidylprolyl isomerase A (*PPIA*) transcript stability remained high after 12 weeks exposure to short photoperiod [7]. But hypothalamic *Hprt* did not show adequate stability across photoperiodic conditions [7]. In those voles, there were no reports of photoperiod dependent changes in abundance. These findings show that *Act* might maintain stability across photoperiod in some plants, but there are species-specific effects in hamsters and voles. Overall, *18s* remains stable across photoperiod conditions and should be used in conjunction with other reference transcripts to normalize target transcript of interest expression. One caveat of *18s* is that the sequence does not possess a polyA tail and so is unsuitable for experiments where cDNA is synthesised using poly(dT) primers. Another caveat is that *18s* is a highly expressed transcript and so may not be suitable for analysis of lowly expressed transcripts.

In addition to photoperiod, tissue type represents a challenge for reference gene selection. In pigs, *Hprt* and *Act* expression has been demonstrated to be highly stable across multiple tissues, while *Gapdh* was unstable and unsuitable as a reference gene [25]. Analyses that used NormFinder on *Hprt* and *18s* expression were also established to be the most stable reference genes in mouse liver and adrenal gland while *Act* and *Gapdh* were less stable [26]. A study in goats investigated 15 tissues and found that *18s* was the most stable gene when all tissues were considered (Normfinder: 0.156), though with some tissue specificity was identified for *Hprt* in kidney (Normfinder 0.051) [27]. *18s* has been shown to be stable across multiple tissues in catfish, while *Gapdh* is showed low stability [28]. In forest tree *Populus* species, *18s* stability by Normfinder is highly dependent on the tissue investigated [10]. These data indicate clear ranking for reference transcripts across trees and vertebrates in which *18s* is consistently found to be stable across tissues, followed by *Hprt*, *Gapdh* and then *Act*. Our data support this ranking as *18s* and *Hprt* were generally the most stable across tissues except in liver and kidney. Since *18s* also showed no significant effect of photoperiod on measures of abundance, this transcript should be selected for molecular analyses in studies of photoperiodism. Indeed, multiple reference transcripts should be used to normalize target of interest transcript expression. Our findings suggest that *Hprt* or *Act* would be highly suitable, depending on the tissue analysed, to determine geometric means when calculating fold changes in expression. Male and female Siberian hamsters display similar response to SD, particularly with regards to body mass. However, studies in Brandt’s voles have identified different optimal reference transcripts for males and females [7]. This study focuses on males, this is due to limitations on the number of animals. It is likely that female animals will display some differences in expression of these transcripts and so a mixed male and female analysis using the same number of animals would be insufficient.

Daily rhythms in reference gene expression may present additional challenges for seasonal molecular analyses. One limitation of this study is the use of a single daily sample and photoperiod-treatment time point (i.e., 12 weeks SD). Previous work in Siberian hamsters has shown that hypothalamic *Gapdh* expression is significantly lower in SD compared to LD hamster shortly after lights exposure (i.e., 1.5hr after lights on) and the onset of the dark (i.e., 4.5hr after lights off) [21]. In the present study, tissue was collected 4-5hrs after lights on and *Gapdh* expression was found to be significantly lower in SD spleen and adipose tissue compared to LD hamsters. Both studies indicate that *Gapdh* may be expressed at higher levels in SD hamster tissue, but the difference might be time of day dependent. Similarly, *Act* expression is unstable across daily time in rat liver [26]. These patterns indicate that time of day sampling must be considered when collected tissue for molecular analyses of photoperiodic responses.

Another limitation of this study is that only male tissue was selected for photoperiod analyses due to the availability of selected samples. The patterns of reference transcript cycling time value noted within this manuscript may be different for female animals and additional work should be carried out for establishing suitability within females. Previous work in Brandt’s voles did not find sex differences in reference transcript cycling time but did identify variation in the optimal reference transcript for quantitative analyses [7]. For both males and female, it is currently unknown whether the reference transcripts show an age-related change in expression. Although age was counterbalanced across the two photoperiods in the present study, in future studies sex and age should be considered as a potential variable for reference gene selection. It is important to also highlight that this investigation used only one primer set for each gene, different primer sets may yield greater stability and efficiency while maintaining abundance [29]. In summary, our study has identified *18s* and *Hprt* as consistent reference genes across tissues for molecular analyses in a photoperiod mammal. Overall, our data provide a foundation to assure precision in qPCR analyses in Siberian hamsters and can be used as a foundation for photoperiodic studies across mammalian species.

## Supporting information

S1 Table Raw data for reference transcript abundance, efficiency, variabiliy and stability across 8 Siberian hamster tissues. (XLSX)
